# ENSO diversity driving low-frequency change in mesoscale activity off Peru and Chile

**DOI:** 10.1038/s41598-020-74762-x

**Published:** 2020-10-21

**Authors:** Carlos Conejero, Boris Dewitte, Véronique Garçon, Joël Sudre, Ivonne Montes

**Affiliations:** 1 Laboratoire d’Etudes en Géophysique et Océanographie Spatiales (LEGOS), Toulouse, France; 2 Centro de Estudios Avanzados en Zonas Áridas (CEAZA), Coquimbo, Chile; 3 Departamento de Biología, Facultad de Ciencias del Mar, Universidad Católica del Norte, Coquimbo, Chile; 4 Millennium Nucleus for Ecology and Sustainable Management of Oceanic Islands (ESMOI), Coquimbo, Chile; 5 Instituto Geofísico del Perú (IGP), Lima, Peru

**Keywords:** Physical oceanography, Atmospheric science

## Abstract

Transient mesoscale oceanic eddies in Eastern Boundary Upwelling Systems are thought to strongly affect key regional scale processes such as ocean heat transport, coastal upwelling and productivity. Understanding how these can be modulated at low-frequency is thus critical to infer their role in the climate system. Here we use 26 years of satellite altimeter data and regional oceanic modeling to investigate the modulation of eddy kinetic energy (EKE) off Peru and Chile by ENSO, the main mode of natural variability in the tropical Pacific. We show that EKE tends to increase during strong Eastern Pacific (EP) El Niño events along the Peruvian coast up to northern Chile and decreases off central Chile, while it is hardly changed during Central Pacific El Niño and La Niña events. However the magnitude of the EKE changes during strong EP El Niño events is not proportional to their strength, with in particular the 1972/1973 El Niño event standing out as an extreme event in terms of EKE increase off Peru reaching an amplitude three times as large as that during the 1997/1998 El Niño event, and the 2015/2016 El Niño having instead a weak impact on EKE. This produces decadal changes in EKE, with a similar pattern than that of strong EP El Niño events, resulting in a significant negative (positive) long-term trend off Peru (central Chile).

## Introduction

In Eastern Boundary Upwelling Systems (EBUS), where the mean circulation is relatively weak, mesoscale eddies tend to play an important role in transporting water masses properties, from heat content to dissolved oxygen^1–4^. It has been shown that the predominant direction of propagation of mesoscale eddies is westward^5^ and the total transport contribution can be comparable to that from large-scale wind and thermohaline driven circulation^6^. They can in particular rectify the mean circulation forming quasi-zonal jets or striations^7,8^, which appears conspicuous in eastern boundary current systems owing to the relatively weak mean circulation^9–11^. In the Southeast Pacific, eddy activity is also suspected to have a significant contribution to the heat budget^3^ with potential feedback to the tropical Pacific climate system considering the sensitivity of large scale winds to sea surface temperature (SST) in the far Eastern Pacific, in particular at El Niño-Southern Oscillation (ENSO) timescale^12,13^. The inability of low-resolution global climate models to account realistically for the eddy heat flux has been invoked as one of the reasons why these models have a persistent warm bias in EBUS^3,14^. Eddy-induced circulation was also shown to be essential for shaping the mean structure and seasonal variability in the oxygen minimum zones (OMZs) for the Southeast Pacific^15,16^ and Northeast Pacific^17^, with also probably important feedbacks to the climate system through their role on the biological pump. Understanding how eddy activity is modulated at climatic timescales is thus important for improving our knowledge of the circulation in EBUS and their role in the climate system, and particularly in the Southeast Pacific that is sensitive to tropical Pacific variability^18,19^ and that hosts one of the largest OMZs in the world^20^.

Eddies in the EBUS are generated by the instability of the coastal current system^21,22^, principally by the Peru–Chile Undercurrent (PCUC) in the Southeast Pacific^22^. There, climatologically forced regional oceanic models were shown to reproduce reasonably well the observed eddy activity as measured by mean eddy kinetic energy (EKE)^3^, indicating that it is dominantly associated with transient propagating eddies impacting the circulation at intraseasonal timescales of variability although eddies themselves can live longer than a few months^23,24^. This is consistent with a modeling study showing a relatively weak sensitivity of eddy activity to intraseasonal equatorial forcing^25^ despite the efficient oceanic teleconnection at such a timescale^26^. This is because equatorially forced intraseasonal fluctuations in coastal currents act as a high-frequency white noise to the instability process, and not as a background change over which instability characteristics (i.e. growth rate and frequency) can vary. At interannual frequency however, there are some evidences that mean EKE could be modulated by the equatorial variability. Chaigneau et al.^23^ show that eddy activity (and the number of generated eddies) has significant interannual variations off the coast of Peru and Chile, although they do not correlate to the NINO3.4 index, i.e. SST anomalies averaged in the region (150°E–150°W; 5°S–5°N), the most commonly used index to measure ENSO strength. Conversely, the modeling study by Combes et al.^27^ found that the generation of subsurface anticyclonic eddies off central Chile is significantly correlated with the ENSO equatorial signal, which is associated with an increase of the PCUC transport. On the other hand, the modeling study of Dewitte et al.^28^, focused on the Peru region, suggested that eddy activity may be influenced by the ENSO amplitude modulation associated with the low-frequency changes in the frequency of occurrence of the two types of El Niño, that is the Eastern Pacific (EP) El Niño with maximum SST anomalies in the eastern equatorial Pacific, and the Central Pacific (CP) or Modoki El Niño with maximum SST anomalies concentrated in the central equatorial Pacific^29–32^. Besides their few numbers, the different methodological approaches and regional focus of these studies call for further investigation of the relationship between eddy activity and climate variability in this region. Here our objective is to revisit the relationship between ENSO, the dominant climate mode in the tropical Pacific, and mean EKE along the coast of Peru and Chile, taking into account the so-called ENSO diversity that broadly refers to the existence of at least two classes of El Niño events, with distinct dynamics^33^ and oceanic teleconnection in the Southeast Pacific^28,34^. We take advantage of 26 years of satellite altimeter data (1993–2018) that has now sampled two strong El Niño events (1997/1998 and 2015/2016), which allows addressing the impact of extreme events and that spans almost three decades, providing also insights on decadal variability. Through high-resolution regional oceanic modeling (ROMS), we are able to expand the time period of investigation over five decades (1958–2008)^28^ and address the modulation of ENSO diversity, which is an expression of the modulation in ENSO amplitude (i.e. moderate vs strong El Niño events) and patterns (CP vs EP) at decadal timescales. Regional model simulations, which include a Control Run (CR) and a sensitivity experiment to boundary forcing (Kelvin), are also used for investigating processes at work and forcing mechanisms.

## Results

### EKE-ENSO relationship during satellite data era

**Figure 1:**
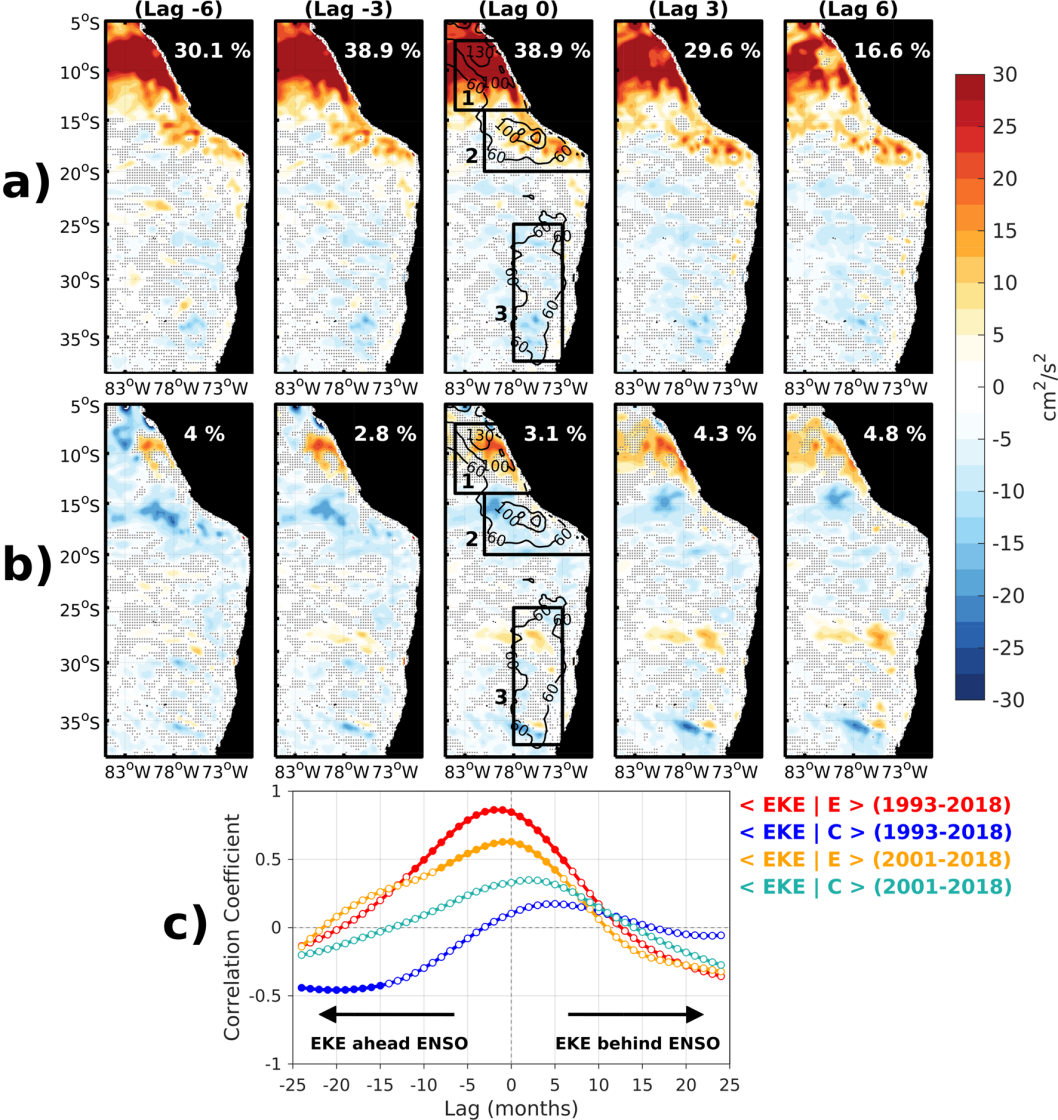
Regression coefficients between interannual EKE variability and the (**a**, upper panels) E index and the (**b**, middle panels) the C index for altimetric observations over the 1993–2018 period at different time lags [negative (positive) lag indicates that EKE is ahead (behind) the ENSO indices in all panels]. The boxes in the panels for lag zero correspond to the regions [northern Peru (box 1), southern Peru (box 2) and offshore central Chile (box 3)] where EKE is averaged for statistics provided in Table S1 (Supplementary Material). The percentage of explained variance averaged over the box 1 is indicated in each panel. Black contour lines (60, 100 and 130 cm^2^/s^2^) in the map for lag zero correspond to the mean EKE values over the whole period. Stippling indicates that regression coefficients are non-significant at the 95% confidence level based on the Student’s *t* test. (**c**) Lagged-correlation between EKE averaged over the box 1 and the ENSO indices, considering different time periods. Full (white) circles indicate that correlation is significant (non-significant) at the 95% confidence level based on the Student’s *t* test. Lag is in month in all panels.

The instantaneous EKE-ENSO relationship is diagnosed from the bilinear regression of interannual EKE variability from satellite altimeter data onto two independent ENSO indices (E and C, see “Methods” section) during the 1993–2018 period. Figure 1 shows the spatial patterns of the lag-regression coefficients indicating where the maximum variation in EKE is located during either EP El Niño events (E index) and CP El Niño or La Niña events (C index). The results indicate that the region of Peru is where the regression coefficient onto the E index is the largest (see box 1 in Fig. 1a), which also corresponds to the peak in explained variance (more than 30%). Off central Chile the change in EKE due to EP El Niño is much weaker and opposite than that over the Peru region (see box 3 in Fig. 1a). 6 months prior to the peak of EP El Niño events, the mesoscale activity increases (decreases) off Peru (central Chile). As EP El Niño events develop, mesoscale activity amplifies offshore and decreases near the coast of Peru. Off central Chile, the weak reduction of EKE persists during the EP El Niño cycle. On the other hand, during CP El Niño events (Fig. 1b), mesoscale activity only increases off the Peruvian coast northward of 14°S. Southward of the Pisco region, around 15°S (see box 2 in Fig. 1b), the mesoscale activity is markedly decreased, with significantly negative values of the regression coefficient. Off central Chile, the relationship between CP El Niño and EKE is weak although a significantly positive value of the regression coefficient can be found between 25°S and 30°S in the offshore region. In order to evaluate the phase relationship between ENSO and EKE, the lagged correlation between EKE, in the region where the regression coefficient is the largest (i.e. off northern Peru, cf. box 1 in Fig. 1a), and the ENSO indices is estimated (Fig. 1c). The latter indicates that EKE is ahead of the ENSO peak by 2 months (r = 0.86) during EP El Niño events. Since the C index accounts for both CP and La Niña events, and that La Niña event tends to follow strong EP El Niño event, EKE reduction appears ahead of the C index by almost two years. If the correlation is performed for the 2001–2018 period, when no La Niña events were preceded by a strong El Niño event, we find that EKE increases during CP El Niño events, although the correlation is not significant at the 95% level based on a student *t* test. On the other hand, the instantaneous conditional correlation between EKE and the C index when C is negative (i.e. during La Niña events) reaches − 0.6 southward of the Pisco region (box 2), which is significant at the 95% level based on a student *t* test (see Table S1). The latter indicated that EKE decreases (increases) during CP El Niño (La Niña) events. These results reveal that EKE is modulated by EP El Niño events more than by CP El Niño or La Niña events. It is interesting to highlight that the strong 1997/1998 El Niño event is the most extreme EP event in the last 30 years, which differs in amplitude with the recently strong 2015/2016 El Niño event (Fig. S1) marked by record-breaking warm anomaly in the central Pacific. The pattern of Fig. 1a tends thus to be representative of EKE changes during the 1997/1998 El Niño events. On the other hand, the spatial pattern of EKE during the 2015/2016 El Niño event resembles that during the 2002/2003 El Niño event (Fig. S1) which is of CP type.

### Interannual EKE variability dominated by EP El Niño events

**Figure 2:**
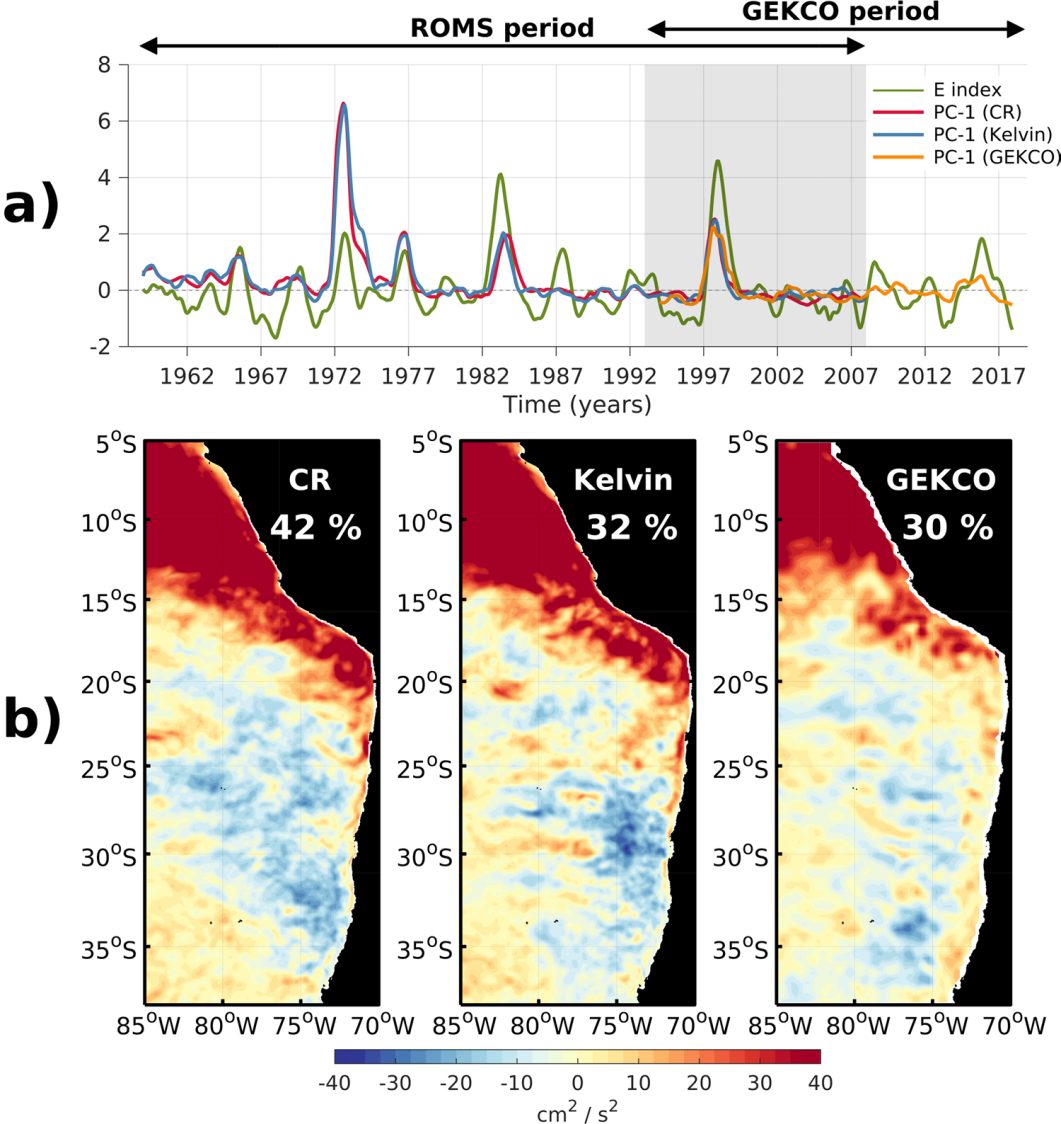
First EOF mode of interannual EKE variability for the model simulations (CR and Kelvin) and the altimetric observations: (**a**) Principal components (PCs) and (**b**) spatial patterns. In (**b**) from left to right, the CR, Kelvin, and altimetric observations. The EOF decomposition of performed over 1958–2008, while satellite data covers 1993–2018 period. EKE anomalies were calculated by removing the seasonal cycle computed over the same period (i.e. 1993–2008) for both satellite data and model simulations, and the pattern for observations was scaled so that CR and observation have an equal standard deviation of PC-1 for during the common period (1993–2008). Explained variance is provided in the maps. Shading in (a) indicates the common period for both observations and model simulations. The green thick line is the E index.

In this section, model results are analyzed. We provide in the supplementary information the material for the assessment of the realism of the simulation used here to extend the observational analysis and investigate processes at work. Overall, our results indicate that despite a tendency of the model to overestimate mean EKE^16,28,34^, the EKE pattern and the EKE–ENSO relationship are rather realistic (see Table S2 and Figs. S2, S3). As a further evaluation of the model skill in accounting for EKE changes at interannual timescales, we present the results of the EOF decomposition for both observations and model simulations (CR and Kelvin, see Methods section) in Fig. 2. The first EOF mode pattern explains comparable percentage of variance (40% for CR and 30% for both Kelvin and altimetric observations) and resembles the projection of EKE onto the EP mode (spatial correlation > 0.8), with EKE increasing off Peru and decreasing off central Chile. The principal components (PCs-1) account to a large extent for EP El Niño events (maximum correlation = 0.55 at lag = 2 months, PC-1 ahead the E index) for both observations and model simulations. Note that similar results were found considering EKE estimated from intraseasonal anomalous currents (see “Methods” section) indicating that these variations do arise from change in eddy activity rather than changes in large scale circulation. A striking feature of the PCs-1 from the model simulations is that the amplitude of the changes is not linearly related to the amplitude of the EP El Niño events accounted for by the E index. In particular, there is a significant change in EKE during the 1972/73 El Niño (almost three times as large than during the 1997/98 El Niño) although this event was not considered as extreme event compared to the three strong events over the period of interest^35^ (i.e. 1982/1983, 1997/1998, 2015/2016). Noteworthy a similar feature is found for the Kelvin simulation, which indicates that this originates from the equatorial oceanic forcing and could be related to specific ocean boundary conditions affecting the stability of the coastal current during the 1972/1973 El Niño (see below).

### Decadal EKE variability

**Figure 3:**
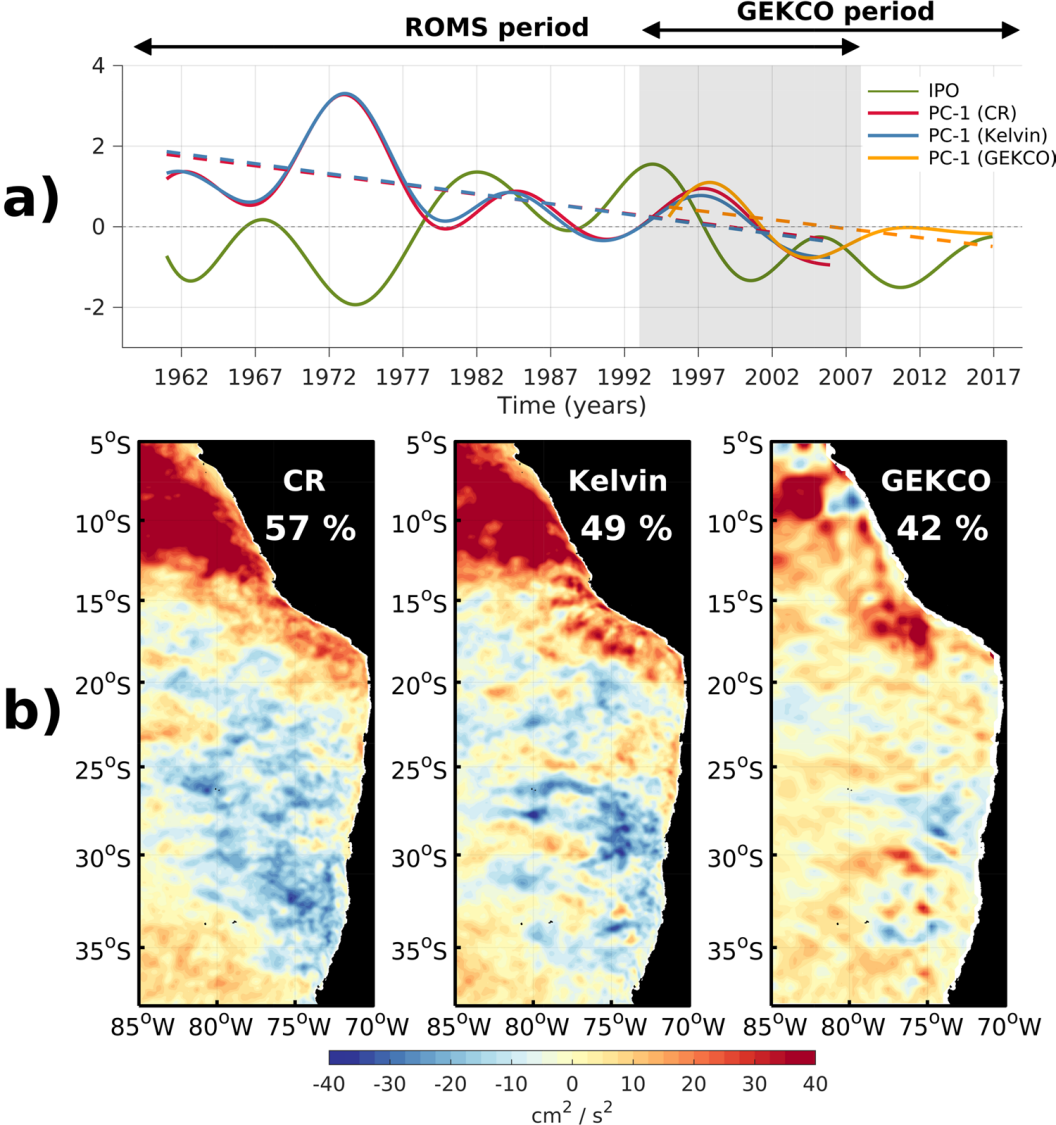
Same as Fig. 2 but for decadal variability. (**a**) Principal components (PCs), and (**b**) spatial patterns. The green line in (**a**) corresponds to the IPO tripole index.

The long-term simulation offers the opportunity to document the decadal EKE variability, and supports the interpretation of the one evidenced from observations which only span 26 years. The dominant mode of decadal EKE variability for both altimetric observations and model simulations are comparable (Fig. 3). The striking feature is that the decadal mode resembles a lot the one for interannual variability in Fig. 2b (spatial correlation = 0.92). It has also a comparable amplitude, with a larger explained variance, which suggests that decadal change in EKE is probably more detectable than interannual changes, despite the fact that the amplitude of decadal SST variability is less than interannual SST variability in the Eastern tropical Pacific. The PC-1 in both CR and Kelvin simulations is associated with a long-term negative trend, which is significant at the 95% level based on a student t-test. Note that current data have been linearly detrended prior to calculating EKE (see “Methods” section) so that such a trend has to emerge from long-term changes in mean EKE. There is a tendency to have an out of phase relationship between PC-1 and the Interdecadal Pacific Oscillation (IPO) (r = − 0.52). Although this is eroded after the climate regime shifts in 1975/76 with a tendency for a quarter-of-a-period phase shift, that is during negative IPO phases, the mesoscale eddy activity reached a maximum (minimum) in the Peru (Chile) region. The amplitude modulation is such that there is a negative trend that results in enhanced (reduced) eddy activity offshore Chile (Peru) in the last decades. Such a tendency is also suggested from observations, which results however from the large amplitude modulation (positive) of EKE during the 1997/1998 El Niño event. The similar results from both CR and Kelvin simulations suggest that the decadal modulation of eddy activity is forced at the oceanic boundaries and may result from the ENSO amplitude modulation or diversity owing to the similar patterns of the modes for interannual and decadal variability. The amplitude in decadal modulation of EKE is somewhat weaker than for interannual timescales over the domain but is as large off Peru. The analysis of the model simulations supports the interpretation of the decadal variability in the observations resulting from a physical process and not from a statistical artifact (due to the shortness of the record). The simulations suggest in particular that the negative trend in PC-1 is influenced by the large amplitude in EKE during the 1972/1973 El Niño event (see also Fig. 2a).

### The 1972/73 El Niño event

**Figure 4:**
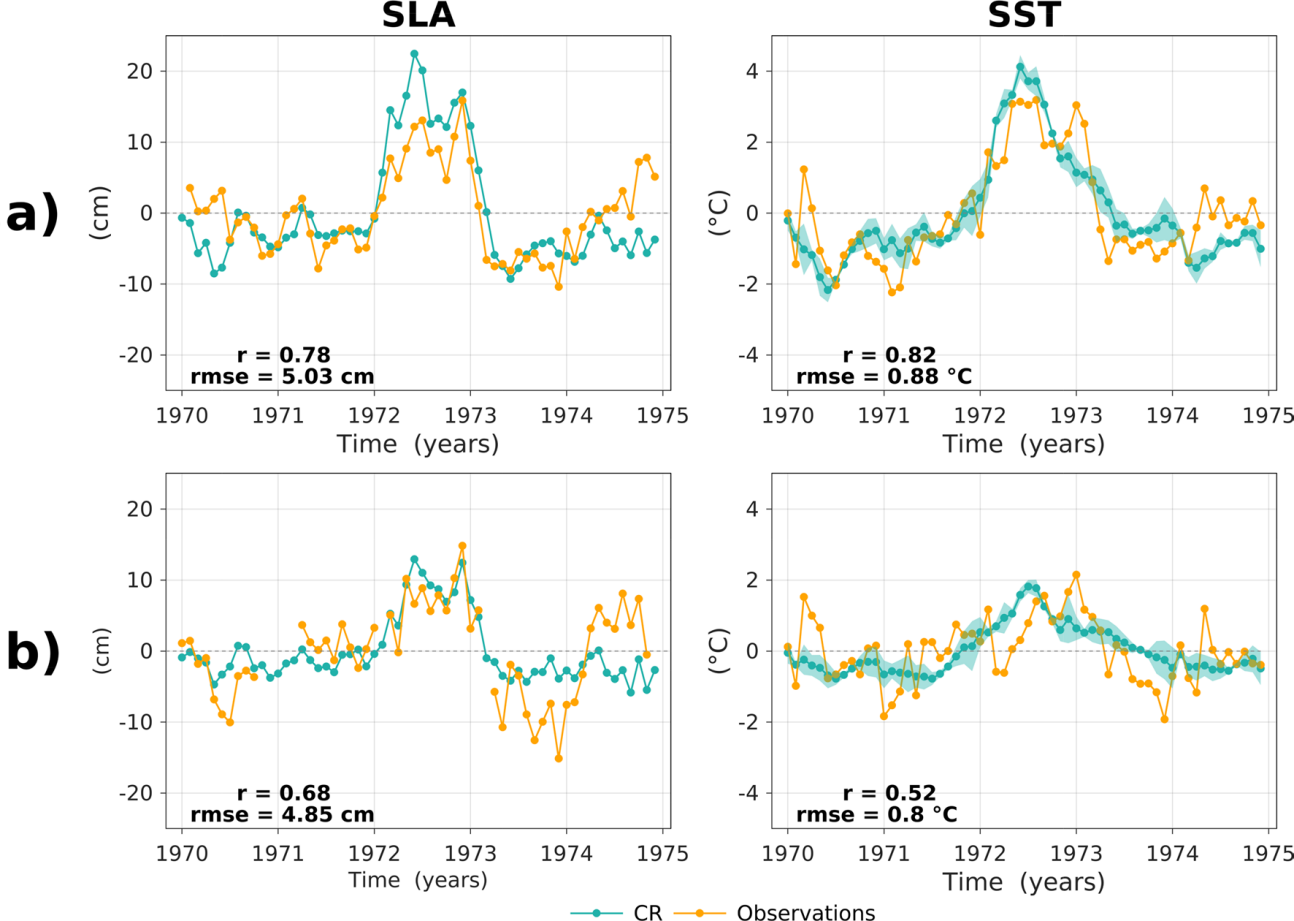
Evolution of sea level (SLA, left panels) and sea surface temperature (SST, right panels) anomalies from observations and the CR simulation over the 1970–1974 period at (**a**) Caldera (12°S) in Peru, and at (**b**) Antofagasta (23°S) in Chile. The correlation (r) and root mean square error (rmse) are indicated in each panel. The seasonal cycle over the period 1970–1974 was removed for both observation and model. The light shading in the curves for model SST anomalies represents the dispersion amongst the 6 values corresponding to the 5-day mean average within a month of the model outputs (i.e. ± the standard deviation amongst these 6 values).

While the 1972/1973 El Niño event can be considered as a strong event since it has a comparable value of the NINO3.4 index than other strong El Niño events (e.g. 1982/1983, 1997/1998 or 2015/2016) that have been recorded in the last five decades, it did not have an Eastern Pacific warming as high as in the 1982/1983 and 1997/1998 El Niño events, with an E value less than half of those years (Fig. 2a, see also Fig. S1). Still it produces the largest changes in EKE off Peru in the model simulations, which questions the extent to which this does not result from a model artifact. We thus provide here some material to support the fact that the large EKE anomalies produced during the 1972/1973 El Niño event do not result either from an unrealistic CR simulation of the circulation along the coast during this event, or from rectification processes within the model domain associated with turbulent dynamics, particularly near the western boundary of the regional model. The evaluation of the model variability was made by using sea level data from tide gauges and in-situ SST observations from HadISST3 data set (Fig. 4, see “Methods” section). The results indicate that the model is realistic and accounts for the magnitude and evolution of the 1972/1973 El Niño event along the coast of Peru and Chile. Further, the analysis of SODA and the CR simulation nearby the western boundary in the equatorial band indicates that the model propagates realistically the boundary condition variability into the model domain. The EOF analysis over the domain (z = [0:400 m], y = [5°S–5°N]) of zonal current anomalies from interannual to decadal variability of both SODA and CR simulation at 88°W (Fig. S4) indicates in particular a very good agreement between both the regional model solution and SODA, with the correlation of the dominant PCs reaching 0.95 and the rms difference between the spatial patterns smaller than 6 cm/s.

Whether or not the SODA data accounts realistically for the subsurface circulation in the eastern equatorial Pacific during the 1972/1973 El Niño event would need further investigation, which is difficult to address owing to the paucity of data during that period. Noteworthy similar amplitude and pattern of the zonal current anomalies in 1972/1973 were obtained from the ORA-S4 reanalysis^36^ (not shown). With such limitation in mind, from our results, we can support the fact that the 1972/1973 El Niño event was associated with a large increase in EKE off Peru, suggesting a non-linear relationship between the El Niño strength as measured by SST indices and the magnitude of eddy activity changes off Peru and northern Chile (see Figs. 1c, S3). Considering the strong oceanic equatorial teleconnection at interannual timescales, essentially through the PCUC^37,38^, such a non-linear response has to originate from details in the fluctuations of the equatorial current systems in the eastern equatorial region impacting the stability of the coastal current systems (see “Discussion”).

### Mechanisms associated with low-frequency EKE variability

**Figure 5:**
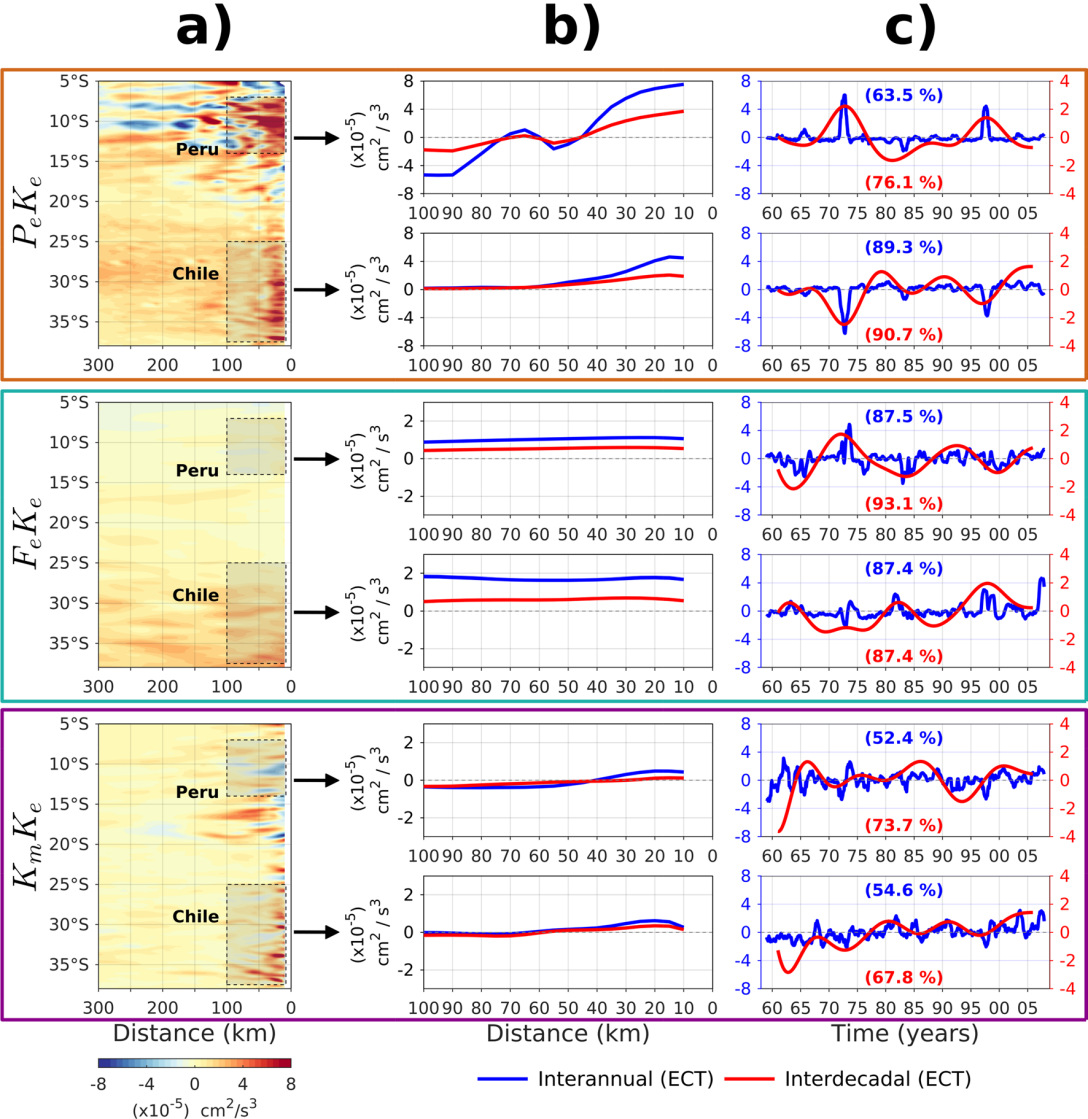
Energy conversion terms: baroclinic instability (P_e_K_e_, Eq. 3, upper panels), wind work (F_e_K_e_, Eq. 1, middle panels), and barotropic instability (K_m_K_e_, Eq. 2, lower panels) from the CR simulation over the 1958–2008 period. (**a**) Offshore distance-latitude diagram of mean values over the whole period. The coastline is considered from the 100 m isopleth, which corresponds to the vertical integration of K_m_K_e_ and P_e_K_e_. (**b**) First EOF mode profiles of the energy conversion terms average over the Peru and Chile regions (see rectangles in light shading in (**a**) for the latitudinal range of the considered regions) for interannual (blue curve) and decadal (red curve) timescales. (**c**) Associated principal components. Explained variance is provided in (**c**). Note the different scales in (**b**) for P_e_K_e_ (− 8 to 8) and F_e_K_e_/K_m_K_e_ (− 3 to 3).

As a support to the physical interpretation of the above results, the main terms of the EKE budget (see “Methods” section) were analyzed in a similar manner than for EKE. We present a section as a function of distance from the coast to offshore and latitude of the mean values of the tendency terms (Fig. 5a) in order to emphasize regional differences in their relative contributions. Figure 5a indicates that wind work (F_e_K_e_, Eq. 1) has a weak contribution to the eddy generation off Peru and central Chile, in contrast with the barotropic (K_m_K_e_, Eq. 2) or baroclinic (P_e_K_e_, Eq. 3) instabilities that contribute the most to EKE change in the Southeast Pacific^25^, which is consistent with both CR and Kelvin simulations exhibiting very similar results at least off Peru and northern Chile. Maximum mean values of K_m_K_e_ and P_e_K_e_ are confined in the first 100–150 km offshore and decay westward, with two contrasted regions where their relative contribution varies meridionally: the Peru region (between 17°S–7°S) and central Chile (37°S–25°S). In the Peru region, the P_e_K_e_ appears to be the dominant EKE generation term whereas in central Chile the three energy conversion terms have a comparable contribution. To document their low-frequency variations, an EOF analysis is performed separately over these two regions, which provides coastal profiles as dominant mode patterns for both interannual and decadal timescales (Fig. 5b,c). In terms of spatial pattern and time-evolution, it is clear that EKE variations are controlled to a large extent by P_e_K_e_ at both timescales, that exhibit the largest amplitude near the coast in both the Peru and Chile regions, meanwhile F_e_K_e_ and K_m_K_e_ have a much weaker amplitude (Fig. 5b). Baroclinic instabilities are generated in the first 50 km offshore, with the largest amplitude variability at interannual timescales. Note that during strong EP El Niño events the EKE increases over the Peru region, while it decreases in the central Chile. In some cases (e.g. 1972/1973 or 1997/1998), the changes are such that they impact the decadal variability and long-term tendency in EKE. This is evidenced from the results of the EOF analysis of EKE from both altimetric observations and model simulations (Figs. 2, 3) which show that both the 1972/1973 and 1997/1998 El Niño events yield a long-term negative (positive) tendency in EKE off Peru (Chile). Moreover, the analysis of the lagged-correlation between the PCs-1 of eddy conversions terms and the PC-1 of EKE at interannual to decadal variability, shows that the maximum positive/negative correlations (|r|> 0.5) are associated with the baroclinic instability process (Fig. S5). Thus, P_e_K_e_ fluctuations appear as the main driver of EKE changes at interannual to decadal variability in the Southeast Pacific.

### Non-linearity of the EKE–ENSO relationship

**Figure 6:**
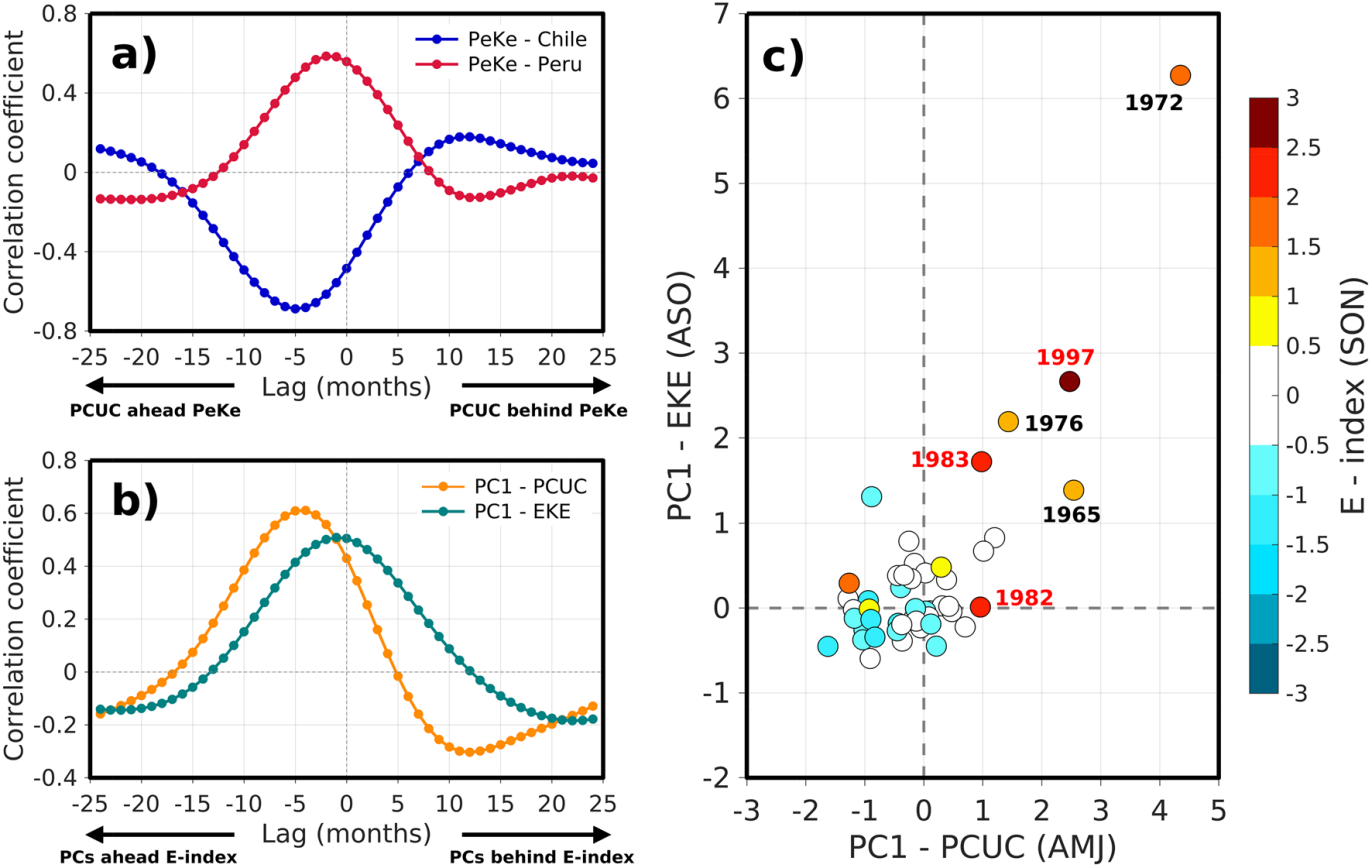
(**a**) Lagged-correlation between PCs of the PCUC transport (cf. Fig. S6a) and baroclinic instability (P_e_K_e_, cf. Fig. 5c) for the Peru (red) and Chile (blue) regions at interannual timescale. (**b**) Lagged-correlation between the E-index and PCs of the PCUC transport and EKE (cf. Fig. 2a). Lag is in months in both (**a**–**b**) panels. (**c**) Scatterplot of PC1 for the PCUC transport (AMJ: April–May–June) against PC1 for EKE (ASO: August–September–October). The selection of the 3-month period for averaging the PCs is based on (**b**) taking the lag that maximizes the correlation with a reference season in SON (September–October–November) that corresponds to the peak climatological variance for the E-index. Colour dots indicate the magnitude of the E index (color bar). The EP El Niño years are indicated near the dots. See text for the method to derive the PCUC transport.

Since baroclinic instability is generally associated with changes in the vertical shear of the coastal system currents in EBUS^21,22^, it is worth documenting the PCUC transport along the coast that acts as a source of vorticity and can produce P_e_K_e_ changes during EP El Niño event (Fig. 6). We find that the PCUC variability (as inferred from the PC-1 timeseries of the PCUC core along the coast, Fig. S6) is highly correlated (anti-correlated) with the PC1-P_e_K_e_ over the Peruvian (Chilean) region, with PC1-PCUC ahead PC1-P_e_K_e_ by ~ 3–5 months (maximum correlation larger than 0.5) (Fig. 6a). The phase shift relationship between PCUC transport and P_e_K_e_ can be interpreted as resulting from the increase in PCUC during the recharge process of ENSO that peaks around 5–6 months prior to the culmination of El Niño^38^ (Fig. 6b). The dominant EOF pattern for the PCUC along the coast (Fig. S6) indicates that the PCUC increases everywhere during the development of ENSO, with however significant latitudinal variability. In particular, two regions of maximum poleward PCUC transport at ~ 14°S and ~ 19°S coincide with the local maximum in EKE changes (Fig. 2), meanwhile in central Chile the PCUC transport is reduced. Note that the PCs-1 of PCUC transport and zonal currents around the equator at 88°W (see Figs. S4, S6) are highly correlated (r > 0.70 which is significant at the 95% level based on a student t-test) at both interannual and decadal timescales and that the rms difference of the two timeseries is weak (~ 70% of the mean rms), indicating that the PCUC transport changes are linearly associated with the changes in the equatorial circulation from the Equatorial Undercurrent (EUC) transport. The relationship between EKE changes and the E index is however highly non-linear (Fig. 6c). In particular, the 1972/73 El Niño event experiences a change in the amplitude of the PC1-EKE and PC1-PCUC that is 363.8% and 443.9% (235.4% and 175.9%) larger than that of the 1982/83 (1997/98) El Niño event, respectively, despite much weaker value of the E index (Fig. 6c). Besides, the regression coefficient between both the E index and the PC-1 for EKE is very sensitive to whether or not we exclude one of the three strong EP El Niño events from the analysis. Variations in the regression coefficient reach 60% of the mean values.

## Discussion

In this paper, we documented the modulation of mesoscale activity in the Southeast Pacific at ENSO timescales through observations and long-term numerical simulations. The satellite altimeter data have now sampled two strong El Niño events, which allows getting insight into the relationship between ENSO and eddy activity. Here we address this issue aided by a previous modeling work dedicated to producing a realistic regional long-term simulation of the Southeast Pacific^28^, overcoming in particular limitations in atmospheric forcing products^39,40^. The study also takes into account the ENSO diversity^33^ that refers to the existence of two types of El Niño events with distinct spatial pattern and amplitude^31^. It is shown that the interannual EKE variability is mostly associated with strong EP El Niño events. The latter yields an enhanced (reduced) mesoscale activity off Peru and northern Chile (central Chile). On the other hand, during CP El Niño (La Niña) events, the mesoscale activity is hardly changed, with a weak increase (decrease) off Peru and a reduction (increase) southward of the Pisco region (15°S). While model results are consistent with our findings from altimetry, they also indicate that the EKE-ENSO relationship arises mostly from the oceanic teleconnections since the model simulation with steady atmospheric forcing (Kelvin) yields comparable results. This is consistent with ENSO-related equatorial Kelvin wave propagating along the coast modulating coastal current stability and stratification. In particular, this oceanic teleconnection is distinct for EP and CP El Niño events, which explains why CP El Niño events are not influential in EKE. First the oceanic teleconnection during CP El Niño event is much less energetic than during EP El Niño which is reflected into the magnitude of SST anomalies along the coast of Peru^28^. This is due to the fact that the energy of the equatorial Kelvin waves during CP El Niño is less than during EP El Niño and they cannot propagate far into the eastern equatorial Pacific, because they encounter a density front near 120°W along the equator associated with the sloping thermocline and dissipate there^41^. Second, the energy of equatorial Kelvin waves during CP El Niño events peaks in the intraseasonal frequency band (i.e., periods between 30 and 90 days) so that they are trapped along the coast, conversely to equatorial Kelvin waves at interannual timescales that radiate as extra-tropical Rossby waves once they reach the South-American coast^26^. This trapping makes that they are much less influential on the baroclinic instability process^25^. This interpretation is confirmed by the results of the EKE budget that indicates that the baroclinic instabilities are modulated at interannual to decadal timescales. The results also suggest a significant non-linearity in the relationship between the amplitude of ENSO (as measured by SST anomalies) and the EKE changes. For example, the model suggests that the 1972/1973 El Niño yielded the strongest EKE change off Peru, while the altimeter observations indicate that EKE was weakly impacted during the strong 2015/2016 El Niño, although these two events have a comparable amplitude in terms of the E index (Fig. S1a) and had a comparable seasonal evolution^35^. Large EKE changes during the 1972/1973 and 1997/1998 El Niño events also appear to rectify on the decadal variability (Fig. 5c), yielding a negative (positive) long-term tendency in EKE off Peru (central Chile). This points out to the likely importance of details in the dynamics of El Niño events in modulating the eddy flux off the coast of Peru. In particular, the 2015/2016 El Niño event with a comparable value of the NINO3.4 index at its peak phase than previous strong events, which was favored by the build-up of heat content^42^ back to the beginning of 2014, has been classified as a strong EP El Niño event. However it was not as strong in the far Eastern Pacific due to air-sea feedbacks off Peru that damped its growth in the far Eastern Pacific^35^ and its dynamics shared characteristics of that of a CP El Niño event^43^. This may explain its weak impact on EKE and its similarity with CP El Niño event in terms of its impact in EKE off Peru. Another interesting case occurs with the 2017 coastal El Niño event that has recently drawn the interest of the community^44,45^. This event yielded heavy rainfall along the coast of Northern Peru comparable to that of the 1997/1998 El Niño event^46^. This event was characterized by a localized strong warming off the coast of Peru while the central equatorial Pacific experiences near-normal (slightly cooler) conditions. Interestingly, it also was associated to a relatively weak negative EKE anomaly off Peru (see Figs. 2, S7). Model results suggest that this event was triggered by a relatively weak amplitude downwelling oceanic equatorial Kelvin wave combined to anomalous northerly coastal winds^45^. The latter may have resulted from the local atmospheric response to the developing warm coastal SST anomalies, when the climatological southward branch of the intertropical convergence zone starts to develop, i.e. from February-March^45,47,48^. On the other hand, the 1972/1973 El Niño event is associated with a record EKE increase off Peru despite its similarity with the 2015/2016 El Niño event in terms of its magnitude in the far Eastern Pacific^35^. This extreme event in EKE (i.e. 1972/1973) yields a long-term negative trend in EKE off Peru. Similarly, the observational record evidences a significant decreasing trend in EKE due to the large positive anomaly during the 1997/1998 El Niño event. This indicates that strong EP events have the ability to produce decadal changes in EKE not necessarily related to natural mode of decadal variability in the Southeast Pacific like the IPO. We find a weak correlation between the IPO index and the PC-1 timeseries associated with decadal variability in EKE (see Fig. 3a). While the model experiment was not specifically designed to investigate decadal variability, which could explain the relatively weak correlation, our result suggests that decadal variability in EKE could arise from the rectified effect of strong EP El Niño in EKE. The results suggest in particular that strong EP El Niño events that take place every decade or so, can produce energetic “spikes” in the eddy flow through the baroclinic instability that have a residual on the long-term mean (Fig. 5c, top panel), yielding the decadal change in EKE. In other words, EP El Niño events energize EKE decadal variance acting as a red noise process^49^. The reasons why there is a diversity in the response of EKE to strong EP El Niño events would deserve further investigation. At this stage we can speculate that the impact of strong EP El Niño on EKE changes along the coast of South America depends on mean state conditions along the coast of Peru and Chile. In particular, the 1972/1973 El Niño event took place prior to the 1976/1977 climate shift when the mean state was relatively cooler, which may have preconditioned the coastal current system towards being unstable and thus more sensitive to ENSO-induced fluctuations of the PCUC. Although decadal variability in the model simulations maybe biased due to limitations in the atmospheric forcing^39^ that may underestimate in particular the contribution of IPO-induced winds, we find that in both experiments that the baroclinic instability was also reduced from before and after the 1976/1977 climate shifts (i.e. P_e_K_e_ over 1958–1975 is 45.8% (86.2%) larger than over 1978–2008 over the Peru domain in CR (Kelvin) simulation), which is consistent with this hypothesis. We also note that in the regional simulations, the PCUC transport changes during the 1972/1973 El Niño are twice as large as during the 1997/1998 El Niño event (Fig. 6c), which could also explain the large EKE change during that event. This large PCUC transport is associated with a large EUC anomaly at 88°W in the boundary oceanic conditions (Fig. S6) and is ahead the peak of the 1972/1973 El Niño by ~ 2 months. This indicates that the 1972/1973 El Niño event was associated with a significant recharge process, comparable to that of the 1997/1998 El Niño event (Fig. S8), although the processes that yielded the increase in heat content were certainly different^50^. This strong recharge may thus explain the large PCUC anomalies during this particular event observed in the simulations. During the recharge process of ENSO (i.e. 9 to 6 months prior to the ENSO peak), the zonal pressure gradient across the equator is enhanced, which is associated to an overall increase in the equatorial trade winds^51^. This tends to increase the EUC transport which variability is correlated to the PCUC anomaly at seasonal and interannual timescales^37^. However the EUC is not directly connected to the PCUC. Montes et al.^52^ show that in fact the PCUC is predominantly fed by two narrow eastward subsurface currents (also known as Tsuchiya jets) at ~ 3–4°S (primary) and 7–8°S (secondary), which dynamics are highly non-linear^53^. The recharge process also involved non-linear processes^54^ and can be influenced by atmospheric high-frequency noise^55^ so that the relationship between both the ENSO recharge and the EUC, and between the EUC and the PCUC may deviate from linearity.

While we have focused here on surface EKE taking advantage of altimeter data, model studies^27,56^ and *in-situ* observations^24,57^ suggest that subsurface eddies or “Intrathermocline Eddies” (ITEs), also called Puddies^58^, exist in the Southeast Pacific which tend to be anticyclonic. These eddies have a weaker surface signature and are thus not well observed by altimetry, although they represent a significant source of natural variability in the euphotic layer. They are also thought to be connected to the variability of the PCUC^56^, which suggests that their activity could be also modulated by strong EP El Niño events. In particular we find that in the model, EKE off central Chile increases at 200 m during strong EP El Niño (Fig. S9) while it decreases at the surface, suggesting that ITEs are favored during strong El Niño conditions, a result consistent with Combes et al.^27^. This has clear implications for the understanding of the natural variability of the OMZ in the Southeast Pacific since these subsurface eddies, with their core closer to the oxycline, can transport offshore water masses properties and thus modulate the OMZ. Such a process of ENSO-induced variability of the OMZ would deserve further study from the same experimental set up used here^16^, which is planned for future work.

## Methods

### Sea level and sea surface temperature data

We use sea surface height from the GEKCO^59^ (Geostrophic and EKman Current Observatory) product to estimate the surface geostrophic EKE. This data set is based on altimetric data from DUACS (Data Unification and Altimeter Combination System) of AVISO (Archiving, Validation and Interpretation of Satellite Oceanographic data). Data are globally gridded from 1993 to present as daily values with a spatial resolution of 1/4°. We also use sea level anomaly (SLA) data and SST anomalies to validate the model simulation over the period prior to the satellite era. SLA data from tide gauge was provided by the UHSLC (University of Hawaii Sea Level Center), while SST data was obtained from HadSST3^60,61^ (Hadley Centre Global Sea Surface Temperature version 3), which provides monthly uninterpolated SST anomalies in 5° × 5° grid box for 1850-present. The anomalies are relative to a 30-year climatology spanning 1961–1990. HadSST3 is based on *in-situ* measurements of SST from ships and buoys.

### Model simulations

A long-term simulation with the ROMS^62^ (Regional Ocean Modelling System) model is used over the 1958–2008 period developed in Dewitte et al.^28^. This model configuration, which is used as a Control Run (CR) simulation, has a horizontal resolution of 1/12° at the equator, covering the domain extending from 12°N to 40°S, and from the coast to 88°W. 5-daily mean oceanic outputs from SODA (Simple Ocean Data Assimilation, version 2.1.6) provide the open boundary conditions (OBC) for temperature, salinity, horizontal velocity and sea level. To force the regional model at the air/sea interface, wind speed and wind stress from the downscaled product^39^ were used, which was shown to provide realistic forcing from atmospheric reanalysis that generally do not resolve properly near-shore winds^63^. Atmospheric fluxes were derived from the bulk formula using the air temperature from COADS^64^ (Comprehensive Ocean–Atmosphere Data Set) 1° monthly climatology. The reader is invited to refer to Dewitte et al.^28^, Vergara et al.^16,34^, Pizarro-Koth et al.^65^, and to supplementary information for more details on the model description, skill and validation. To evaluate the impact of equatorial forcing onto EKE variability, a sensitivity experiment to atmospheric forcing was performed. This experiment, called Kelvin, consists in using the same oceanic boundary conditions than CR simulation but using a wind forcing corresponding to the 2005 year repeated each year. This 2005 year is selected as a “normal” year in terms of surface atmospheric circulation in the SEP despite the development of a CP El Niño event in 2004. This Kelvin simulation allows assessing the role of the equatorial oceanic forcing on the EKE modulation off the Peru–Chile coasts.

### El Niño and decadal variability indices

The two El Niño indices defined by Takahashi et al.^31^ are used, that are based in the principal components time series associated with the first two EOF modes of the monthly mean SST anomalies in the tropical Pacific (see Fig. S1a). The SST data are from the ERSST.v3b^66^ (Extended Reconstructed Sea Surface Temperature) product of NOAA (National Oceanic and Atmospheric Administration) over the 1958–2018 period. The two uncorrelated indices are well suited for describing the so-called ENSO diversity^33^ by accounting for the variability of Eastern Pacific El Niño (E-index) and Central Pacific El Niño and/or La Niña (C-index). In addition, the Tripole Index for the Interdecadal Pacific Oscillation (IPO) was obtained from NOAA/ERSST.v3b over the same period (see Fig. S1a). The index is based on the difference between the SST anomalies averaged over the central equatorial Pacific and the average of the SST anomalies in the Northwest and Southwest Pacific^67^. Note that both ENSO and IPO indices obtained from observations are very close to those obtained from SODA reanalysis (correlation is above 0.9 for all indices), which was used as an OBC in the regional model.

### Quantifying variability in eddy activity

Most of the existing literature on mesoscale activity in the Southeast Pacific has used geometric methods to count the number of mesoscale structure either directly from dynamic height data or estimate of vorticity^5,23,68^. While these studies provide key information on eddy activity, methodological^69^ and observational^70^ limitations remain. Here, for simplicity and to avoid comparing different methodological approaches, mesoscale activity is measured from the estimate of EKE (estimated as EKE = (u′_g_^2^ + v′_g_^2^)/2), which represents a physically-based metrics that obeys a tracer equation and thus can be objectively interpreted from theory. The EKE was obtained from 5-days detrended surface geostrophic currents anomalies (u′_g_, v′_g_), which are derived from sea surface height. Interannual anomalies were computed from 5-days mean outputs removing the monthly climatology interpolated at 5-days temporal resolution. Then, mean EKE over 3-months running windows followed by a monthly average is estimated, providing series of monthly “mean EKE”, which we refer as just EKE. The seasonal cycle from monthly EKE was further removed to derive EKE anomalies. Intraseasonal anomalies in the velocity field were also considered to derive EKE anomalies to filter out the contribution of changes in the circulation at interannual timescale. The intraseasonal anomalies consist of the departure from the monthly mean following previous studies^71,72^. In either case, a 1-year running mean filter is further applied on the resulting anomalies to filter out remaining high-frequency variations associated with the low-frequency modulation of the seasonal cycle. The obtained interannual EKE field is then analyzed through regressing on indices and/or EOF decomposition. For addressing decadal EKE variability, a butterworth filter (order 5 with a 10 years cut-off period) is used.

### EKE budget

The contribution of each EKE generation process is evaluated from the regional model based on the tracer equation for EKE. We focus here on the energy conversion terms relevant to nearshore following previous studies^21,25^: wind work (F_e_K_e_, Eq. 1), barotropic instability (K_m_K_e_, Eq. 2) and baroclinic instability (P_e_K_e_, Eq. 3). F_e_K_e_ represents the transfer of energy from surface wind-forcing anomalies to EKE, K_m_K_e_ corresponds to the barotropic conversion of mean kinetic energy (K_m_) into EKE, and P_e_K_e_ corresponds to the baroclinic conversion of eddy potential energy (P_e_) into EKE. From the horizontal equation of motion, one can derive the expressions:

1 $${F}_{e}{K}_{e}=\langle \frac{1}{H{\rho }_{0}}\left(u{^{\prime}}{\tau }_{x}{^{\prime}}+v{^{\prime}}{\tau }_{y}{^{\prime}}\right)\rangle $$

2 $${K}_{m}{K}_{e}=\langle \frac{-1}{H}{\int }_{H}^{0}\left(u{^{\prime}}u{^{\prime}}\frac{\partial \overline{u}}{\partial x}+u{^{\prime}}v{^{\prime}}\frac{\partial \overline{u}}{\partial y}+u{^{\prime}}w{^{\prime}}\frac{\partial \overline{u}}{\partial z}+v{^{\prime}}u{^{\prime}}\frac{\partial \overline{v}}{\partial x}+v{^{\prime}}v{^{\prime}}\frac{\partial \overline{v}}{\partial y}+v{^{\prime}}w{^{\prime}}\frac{\partial \overline{v}}{\partial z}\right)dz\rangle $$

3 $${P}_{e}{K}_{e}=\langle \frac{-g}{H{\rho }_{0}}{\int }_{H}^{0}\left(\rho {^{\prime}}w{^{\prime}}\right)dz\rangle $$

where wind stress (τ_x_,τ_y_), velocity field (u,v,w) and density (ρ) variables with prime stand for the anomalies relative to the mean climatology (overbar) calculated over the 1958–2008 period. Since we are interested here in the modulation of these terms at interannual timescales, the brackets stand for the mean over a 3-month running windows to capture transient eddy effect on EKE rate of change. For methodological consistency, anomalies were then calculated in a similar manner than EKE. The constant variables are the gravitational acceleration (g = 9.8196 m/s^2^) and the reference water density (ρ_0_ = 1025 g/L). K_m_K_e_ and P_e_K_e_ were integrated over the surface layer depth (H = 100 m), where the instabilities in the Southeast Pacific are confined^3,25^ and can be directly comparable to wind work.

### Peru–Chile undercurrent (PCUC) transport

The integrated poleward transport of the PCUC was calculated considering the average of along-shore southward current over the area coinciding with the mean position of the PCUC, as inferred from cross-shelf sections along the coasts (see Fig. S10). We considered a rectangular box approximating the vertical extension of the PCUC ranging from 60 to 400 m depth, and its cross-shelf extension, spanning from the coast to ~ 100 km. Furthermore, current speeds smaller than 2 cm/s were not considered in the poleward transport calculation. The use of slightly different boxes size did not change the variability of the transport.

## Supplementary information

Supplementary Information.
