# Supplementary material for: ENSO diversity driving low-frequency change in mesoscale activity off Peru and Chile
Source: Sci Rep. 2020 Oct 21;10:17902. doi: 10.1038/s41598-020-74762-x (PMC7578051; doi:10.1038/s41598-020-74762-x)
Supplement: Supplementary file 1 — Supplementary Information. [file 41598_2020_74762_MOESM1_ESM.pdf]

Supplementary Information for:

ENSO diversity driving low-frequency change in mesoscale activity off Peru and Chile

Carlos Conejero[1*], Boris Dewitte[1,2,3,4], Véronique Garçon[1], Joël Sudre[1], and Ivonne Montes[5]

[1] Laboratoire d'Etudes en Géophysique et Océanographie Spatiales (LEGOS), Toulouse, France.

[2] Centro de Estudios Avanzados en Zonas Áridas (CEAZA), Coquimbo, Chile.

[3] Departamento de Biología, Facultad de Ciencias del Mar, Universidad Católica del Norte, Coquimbo, Chile.

[4] Millennium Nucleus for Ecology and Sustainable Management of Oceanic Islands (ESMOI), Coquimbo, Chile.

[5] Instituto Geofísico del Perú (IGP), Lima, Perú.

* Corresponding author e-mail: carlos.conejero@legos.obs-mip.fr

Table S1. Conditional correlation between interannual EKE variability from satellite altimeter data and ENSO indices over different phases (i.e. E and C indices either positive or negative) and time periods. The EKE was averaged over different regions in the Southeast Pacific (see boxes in the middle panels of Figure 1a). Bold numbers indicate that correlation is significant at the 95% confidence level based on the Student's t-test.

**ENSO Phase**	**< EKE | E > 1993-2018**	**< EKE | C > 1993-2018**	**< EKE | E > 2001-2018**	**< EKE | C > 2001-2018**
Box 1: Positive (E, C > 0)	**0.88**	0.04	**0.48**	**0.56**
Box 1: Negative (E, C < 0)	**0.49**	- 0.22	**0.56**	- 0.36
Box 2: Positive (E, C > 0)	**0.67**	- 0.31	- 0.18	- 0.21
Box 2: Negative (E, C < 0)	0.04	**- 0.50**	0.03	**- 0.60**
Box 3: Positive (E, C > 0)	- 0.25	- 0.05	- 0.04	- 0.08
Box 3: Negative (E, C < 0)	- 0.15	- 0.01	0.14	0.08

Table S2. Comparisons of the spatial patterns of the linear regression coefficients of the interannual EKE variability onto the E index (in cm^2/s^2) over the 1993-2008 period (see Figure S2 at lag zero) between satellite altimeter data and the model simulations (CR and Kelvin). SigmaF is the ratio RMS(model)/RMS(altimeter), where RMS is the Root Mean Square, and r is the correlation. RMS difference (diff) corresponds to RMS(altimeter – model).

**Model simulation**	**r**	**RMS (model)**	**RMS (diff)**	**SigmaF**
CR	0.75	21.31	13.9	1.41
Kelvin	0.77	21.45	14.2	1.42

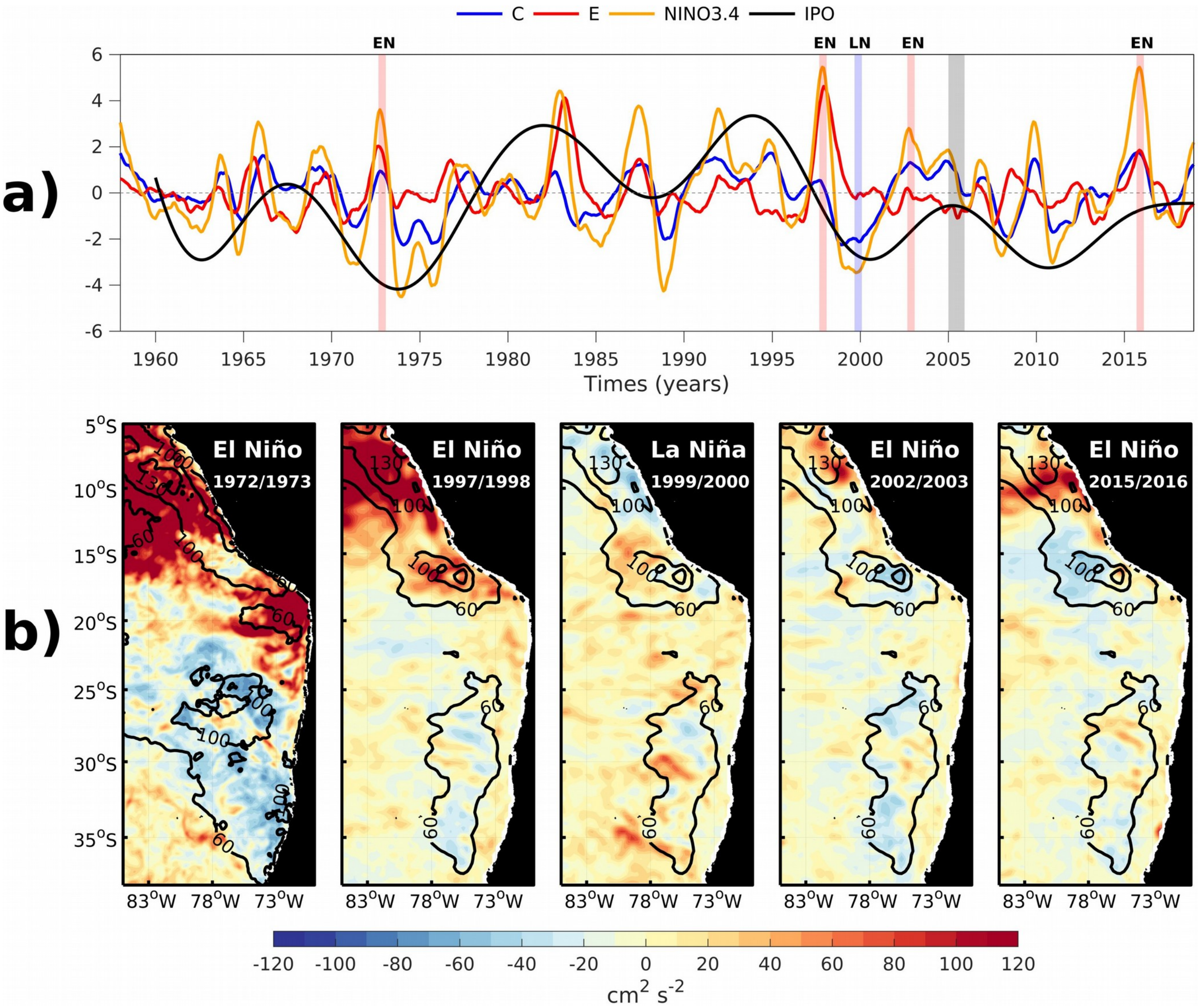

Figure S1. (a) Climate indices (ENSO and IPO) calculated from ERSST.v3b over the 1958-2018 period. (b) EKE anomalies during spring-summer (September to February) of different ENSO events. EKE anomalies during the 1972/1973 El Niño event were calculated from ROMS-CR simulation and during the others events were calculated from satellite altimeter GEKCO (see Methods in main text). Red and light blue shadow colors in (a) correspond to spring-summer period used to different El Niño (EN) and La Niña (LN) events. The gray shading in (a) indicates the 2005 year used as a repetitive atmospheric forcing for Kelvin simulation (see main text), showing that there were no marked anomalous climatic conditions during that particular year. The EKE anomalies were calculated removing the seasonal cycle over the 1993-2018 (1958-2008) period from GEKCO (ROMS). Black contour lines in the maps (60, 100 and 130 cm^2/s^2) correspond to the mean EKE values over the 1993-2018 (1958-2008) period from GEKCO (ROMS).

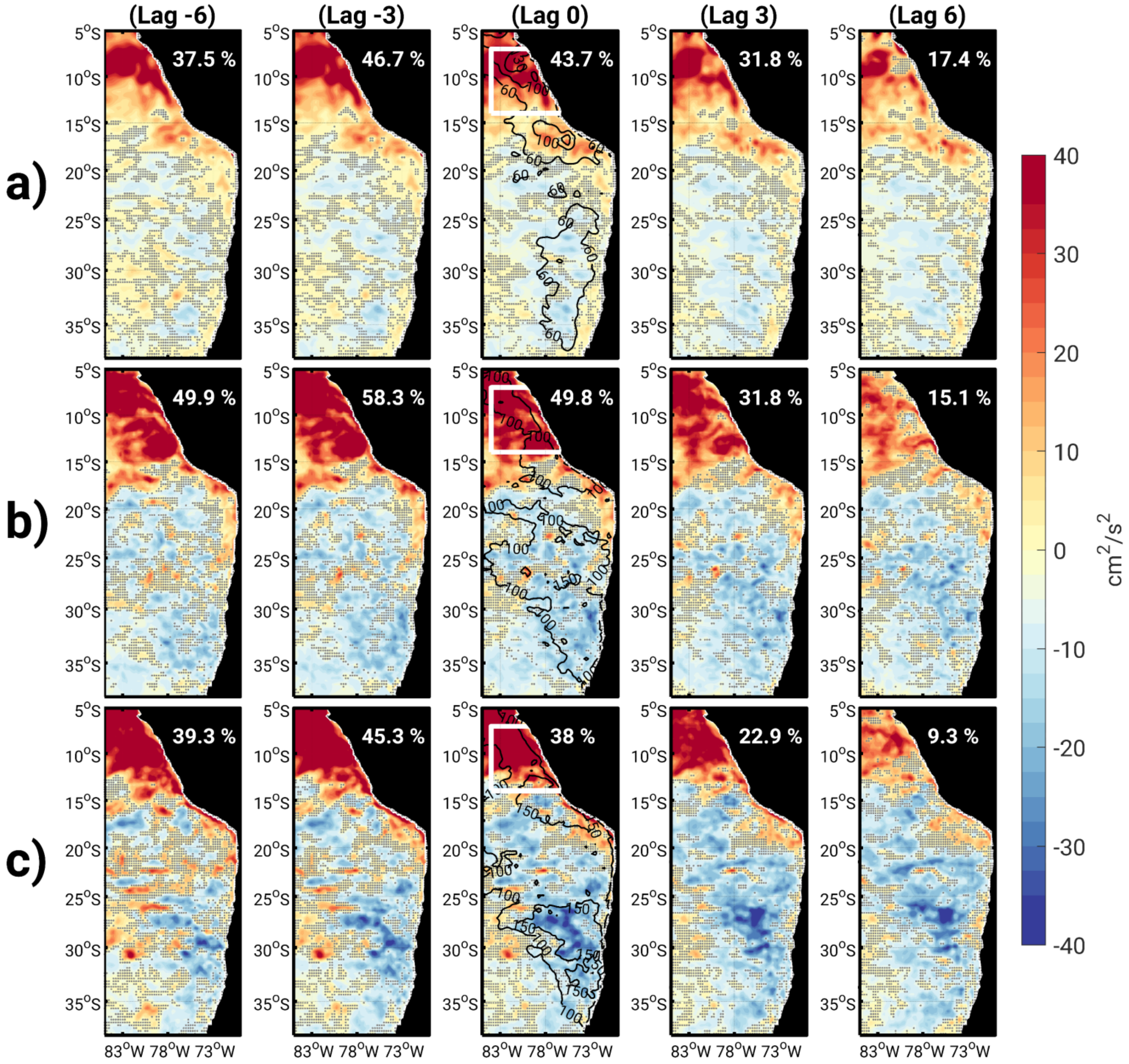

Figure S2. (a) Same as Figure 1a (see main manuscript) but for the analysis performed over the common period between model simulations and observations, i.e. 1993-2008, and (b, c) for the model simulations ((b) CR and (c) Kelvin). The percentage of explained variance averaged over the Peru region (see white box in the maps at lag zero) is indicated on each panel.

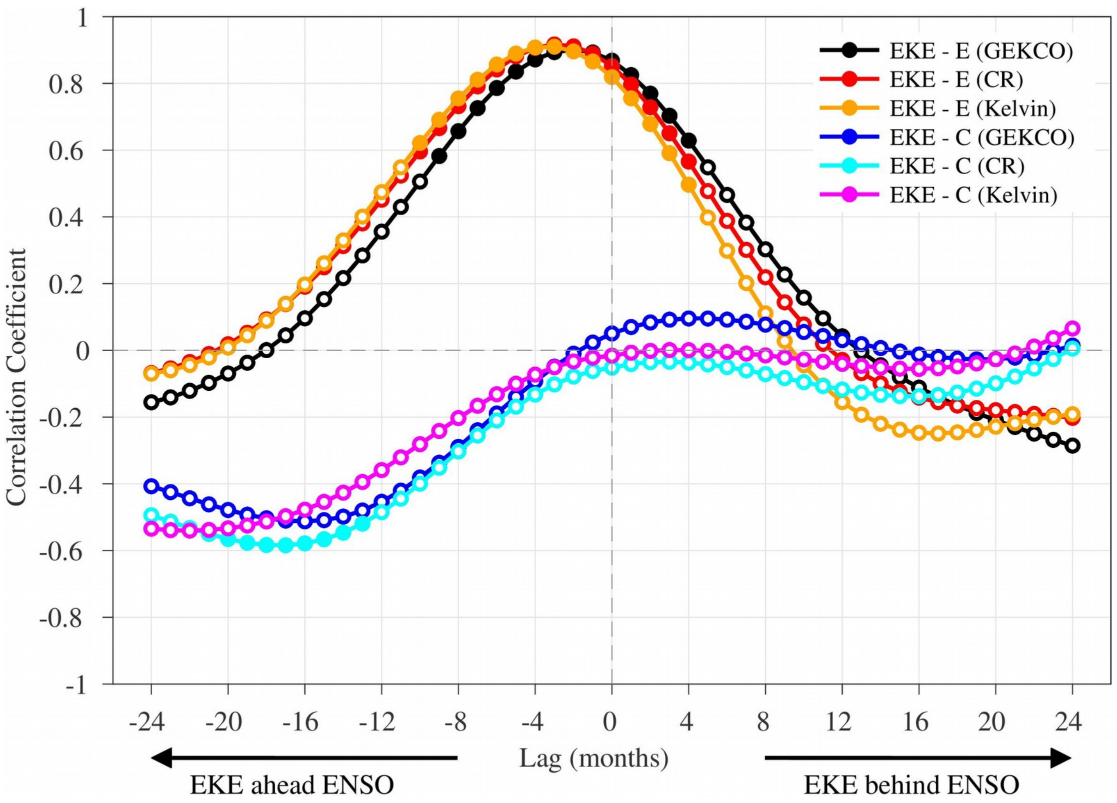

Figure S3. Lag-correlation between interannual EKE anomalies, averaged over the Peru region (14°S-7°S, 83°W-coast), and ENSO indices over the 1993-2008 period. The EKE anomalies were calculated from satellite altimeter data (GEKCO) and model simulations (CR and Kelvin). Full (white) circles indicate that correlation is significant (non-significant) at the 95% confidence level based on the Student's t-test.

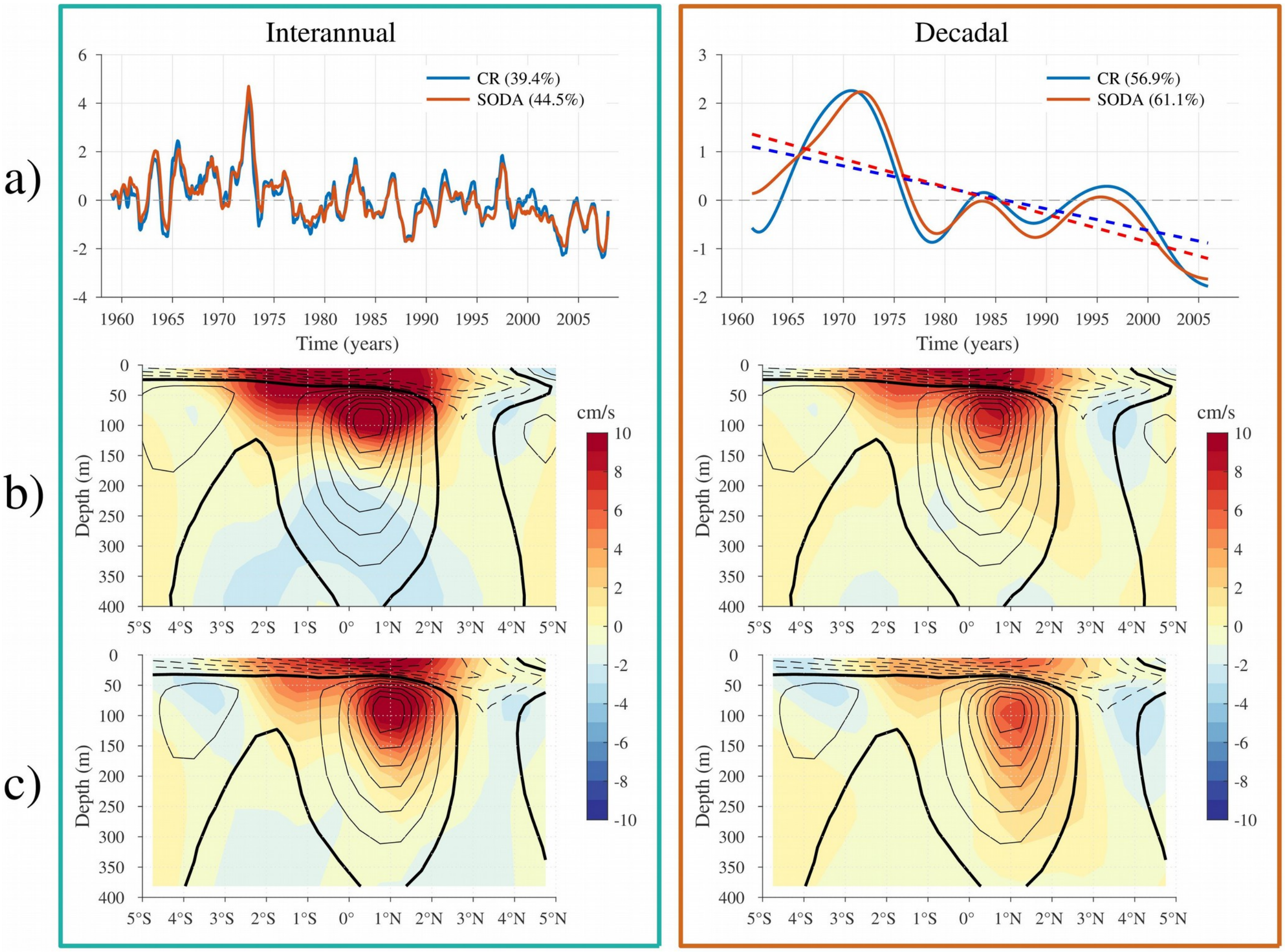

Figure S4. First EOF mode of the low-frequency variability (interannual to decadal) of the zonal currents anomalies at 88°W and over the domain (z=[0:400m], y=[5°S-5°N]) for the CR simulation and SODA, over the 1958-2008 period. a) Principal components, b,c) spatial pattern for b) SODA, and c) CR simulation. Explained variance is provided in a). Solid thick black line in b,c) corresponds to 0 cm/s, and thin (dotted) black lines correspond to positive (negative) eastward (westward) velocities every 2 cm/s. Note that the Equatorial Under Current (EUC) is centered around 0°-1°N and 100 m depth for both CR experiment and SODA. Linear long-term trends are plotted in a) for decadal variability (dashed lines), which are significant at the 95% level based on the Student's t-test.

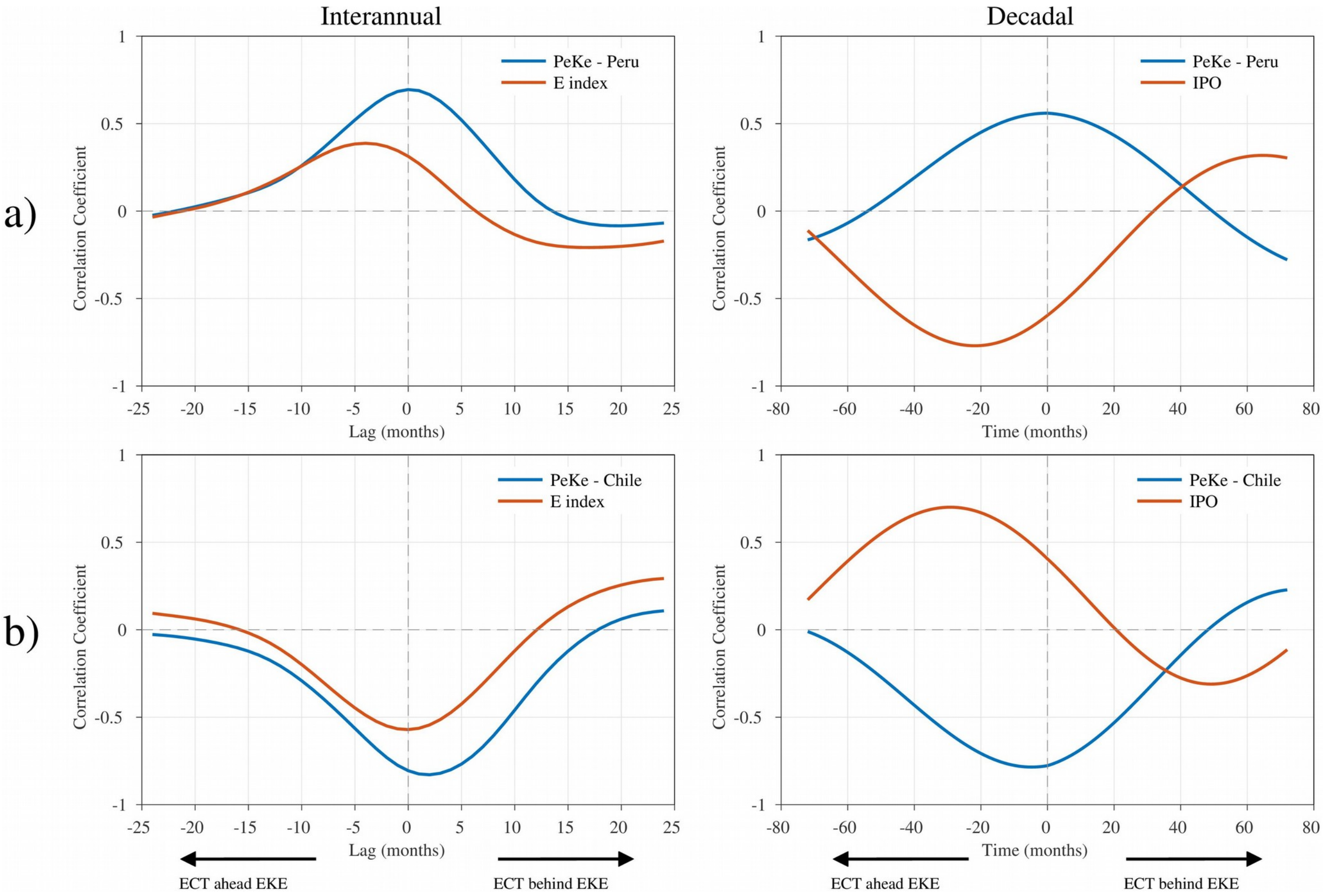

Figure S5. Lag correlation between the PCs-1 of the eddy conversion terms (ECT) and EKE from CR simulation at (left panels) interannual to (right panels) decadal timescales for the a) Peru and b) Chile regions. Note that the maximum/minimum correlations (|r| > 0.5) are associated with the baroclinic instability process.

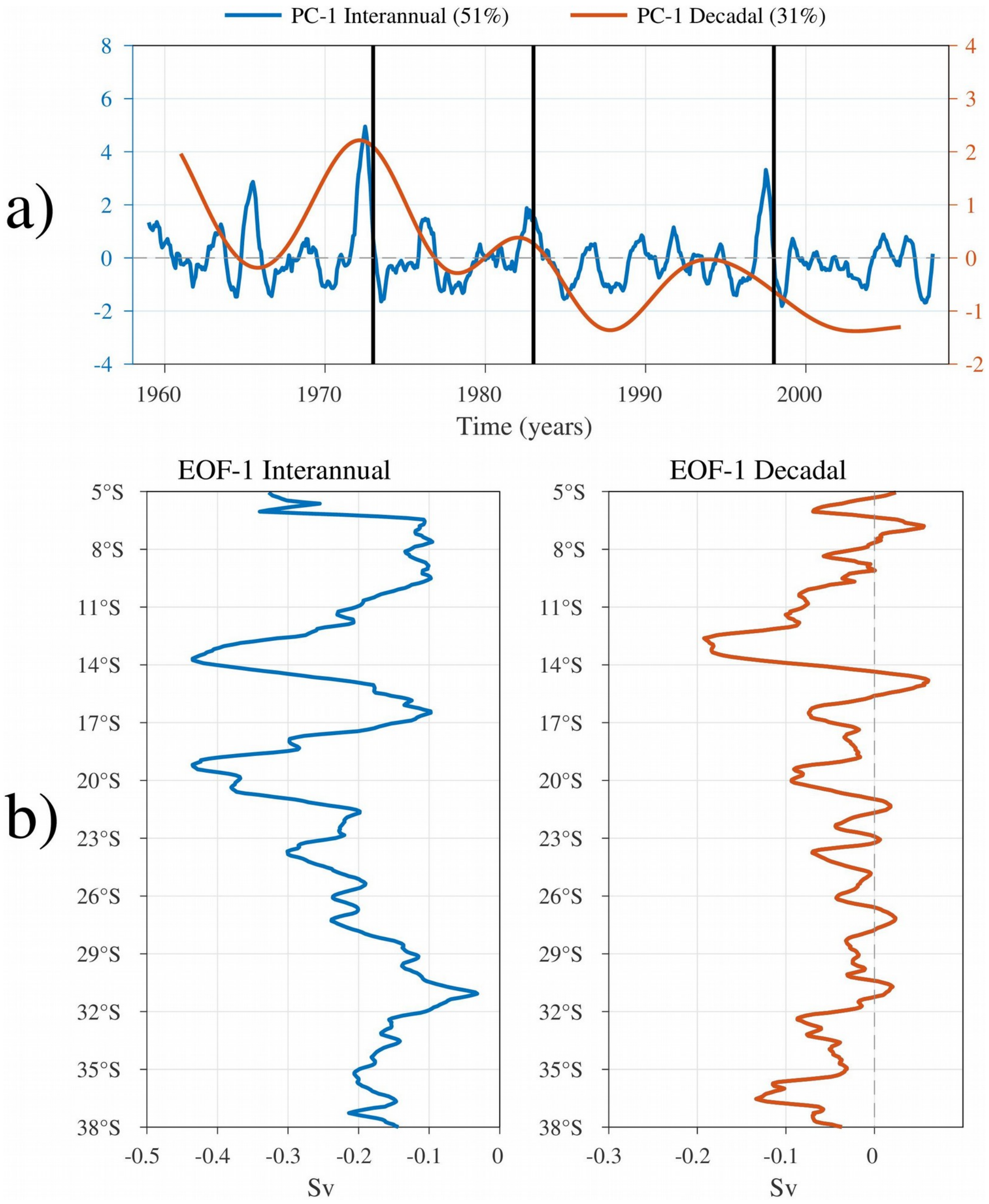

Figure S6. First EOF mode of the Peru-Chile Undercurrent transport (see Methods section) between 5°S and 38°S for interannual (blue) and decadal (red) timescales in the CR simulation over the 1958-2008 period. a) Principal components, b) spatial pattern. Explained variance is provided in a). Black lines in a) represent the peak of El Niño during 1972/73, 1982/83 and 1997/98 events, which corresponds to January of 1973, 1983 and 1998, respectively.

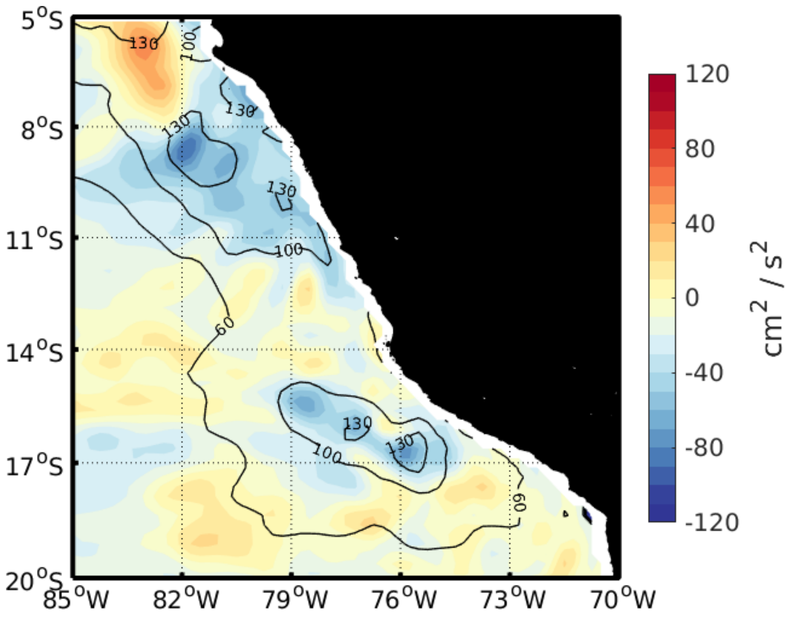

Figure S7. EKE anomalies averaged over the season February-March-April during the 2017 coastal El Niño event from observations (GEKCO). The EKE anomalies were calculated removing the seasonal cycle over the 1993-2018 period. Black contour lines (60, 100 and 130 cm^2/s^2) correspond to the mean EKE values over the 1993-2018 period.

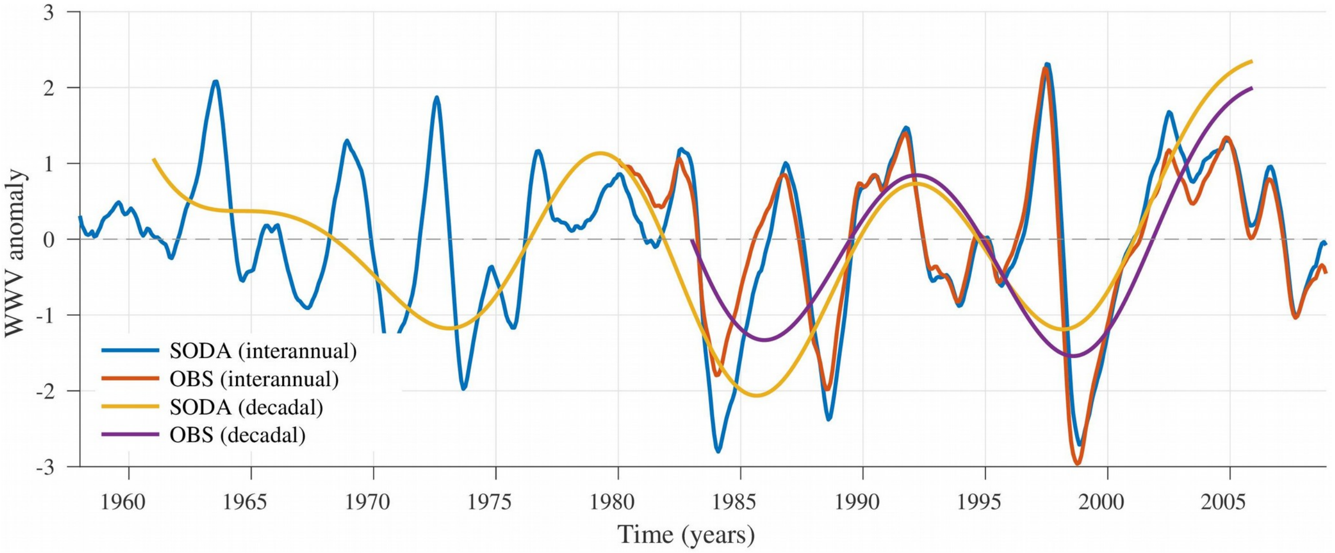

Figure S8. Low-frequency variability of the Warm Water Volume (WWV) from observations and the SODA Reanalysis. The WWV was calculated as the depth averaged temperature over the upper 300 m (T300) in oceanic regions of the equatorial Pacific between 5°N to 5°S, 120°E to 80°W . The WWV observations are from the ocean analyses of the Bureau National Operations Centre (BNOC) at the Australian Bureau of Meteorology, which are based on temperature profiles from TAO moorings, Argo floats and XBTs.

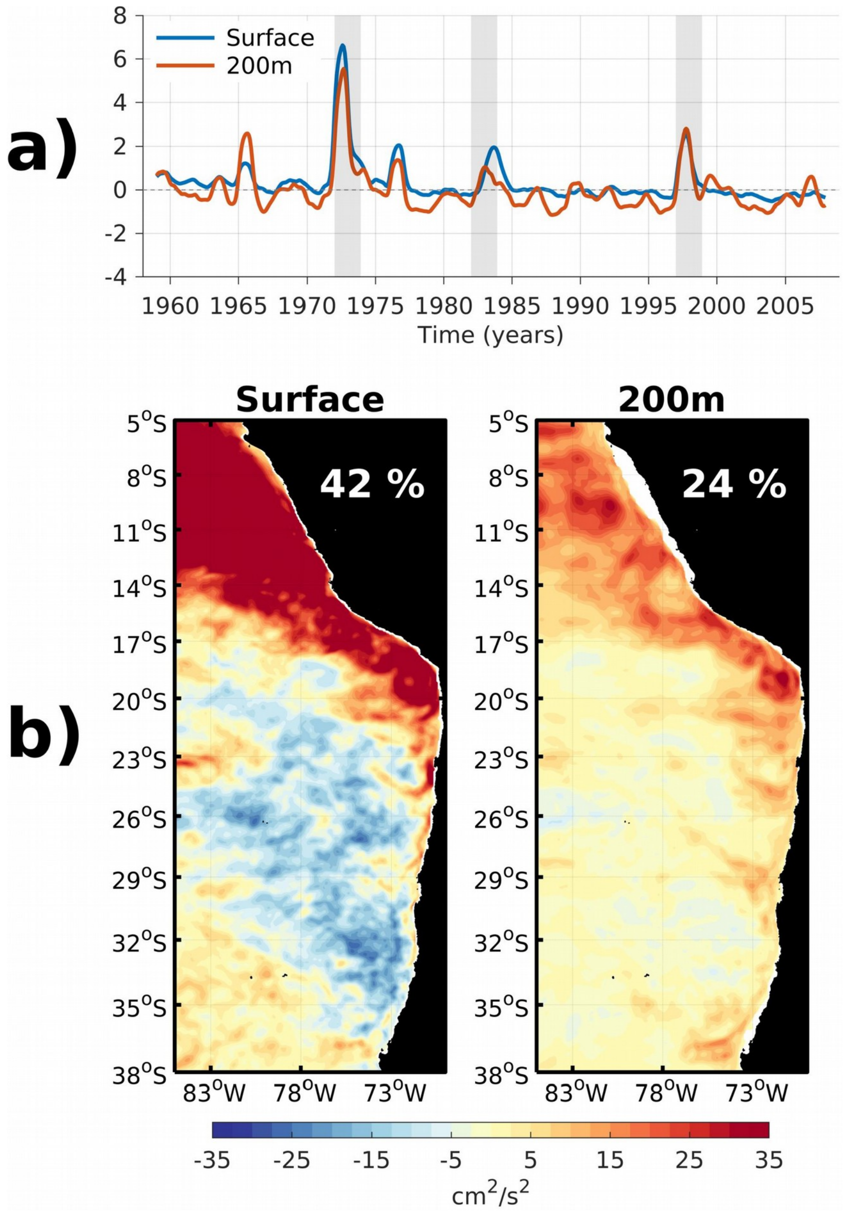

Figure S9. First EOF mode of interannual EKE variability in the Southeast Pacific at the surface and at 200m depth from the CR simulation over the 1958-2008 period. a) Principal components, and b) spatial patterns. Light gray shading in a) corresponds to the three strong EP El Niño events (1972/73, 1982/83 and 1997/98). Explained variance of the EOF modes is provided in the maps.

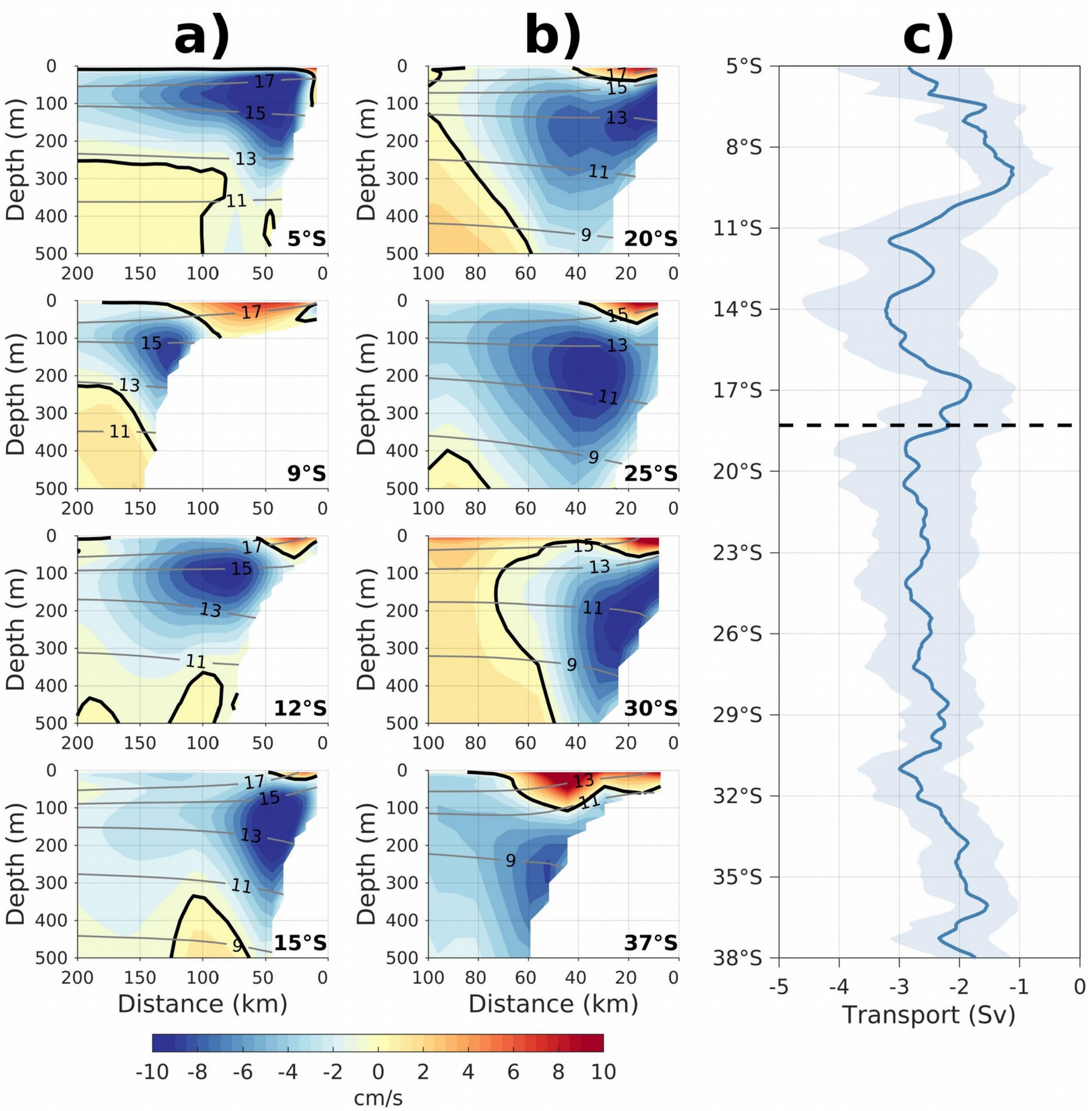

Figure S10. Mean meridional currents from CR simulation over the 1958-2008 period and at different cross-shore sections along the coast of Peru and Chile. a) Peru coast, and b) Chile coast. Equatorward (poleward) currents correspond to positive (negative) values in colors. Gray lines indicate the mean isotherms from 9°C to 17°C. c) Long-term mean value of the simulated Peru-Chile Undercurrent transport (see Methods section). The shading indicates the dispersion, i.e.± the standard deviation of the PCUC transport during the whole period. The horizontal dashed black line corresponds to the limit between Peru (5°S-18°S) and Chile (18°S-38°S) regions. Negative PCUC transport values indicate poleward flux.
